# Understanding the role of village fund and administrative capacity in stunting reduction: Empirical evidence from Indonesia

**DOI:** 10.1371/journal.pone.0262743

**Published:** 2022-01-28

**Authors:** Jul Indra, Khoirunurrofik Khoirunurrofik

**Affiliations:** 1 Graduate Programme in Economics, Faculty of Economics and Business, University of Indonesia, Depok, Indonesia; 2 Research Cluster on Energy Modeling and Regional Economic Analysis (RCEMREA), Faculty of Economics and Business, Universitas Indonesia, Depok, Indonesia; National Autonomous University of Nicaragua: Universidad Nacional Autonoma de Nicaragua, NICARAGUA

## Abstract

The Indonesian government launched various programs to handle stunting cases, including village funds. This paper examined the effects of village fund programs and village apparatuses’ capacities to combat stunting based on aggregate data at the district level in Indonesia. Using descriptive data analysis and fixed effect panel regression, we observed that village fund programs could significantly reduce Indonesia’s stunting prevalence, especially outside Java. It also revealed that the increasing education of regional leaders does not necessarily positively impact leaders’ skills in handling stunting. At the same time, the number of village officials has a statistically significant influence on reducing stunting prevalence. It advised that the village budget fund can support national priorities in tackling the prevalence of stunting. Furthermore, it is essential to build the capacity of the village head for increasing awareness of health activities, especially early prevention of stunting, in addition to an adequate number of officials.

## Introduction

Indonesia still faces nutritional problems that severely impact the quality of human resources. There are concerns about stunting (growth barriers) in toddlers as one of the malnutrition issues. The causes can vary, including economic factors due to the community’s low purchasing power and lack of access to health services. Data from basic health research [1] indicate that Indonesia’s stunting rate is 30.8 percent. For the WHO target, the stunting rate should not exceed 20 percent.

Previous studies have demonstrated that stunting is more associated with socioeconomic factors than genetic factors of individuals or families [2–5]. While genetic factors only slightly affect a person’s height at birth, approximately 4–7%, environmental factors influence 74–87% [6]. Maternal education and integrated health promotion combat stunting [7]. Previous research has focused on the household or individual level (immediate causes), while studies concentrating on the community level (contextual factor) are scarce. No research has studied the role of political economy factors in stunting prevalence, especially in Indonesia. The government’s role is highly required to prevent stunting [8–10].

Stunting is widely recognized as a multisectoral problem requiring strong leadership ranging from central to local and village governments. Ensuring that every village and every household has access to core service packs in all districts/cities is key to reducing stunting in Indonesia. Local governments’ role is set out through the provincial government (e.g., providing technical support and monitoring). The regency/city government helps formulate local policies and set targets for accelerating the reduction of stunting to support national targets). The village government, as the spearhead of the implementation of the program, assists with having the authority to organize and manage activities based on the right of the foundation and using village funds for managing stunting [11, 12]. Furthermore, findings of a recent study on Spending Better to Reduce Stunting in Indonesia conducted by the World Bank revealed that stunting-related services at the regional level, especially villages, are mostly financed by village funds, government-funded health cover (JKN), and assistance from the Ministry of Health [13].

The efforts made by the Government of Indonesia in suppressing cases of stunting need to be supported by all parties, including village fund programs. The Ministry of Finance data show that village funds are worth IDR 268 trillion (USD 19.14 billion) for 2015–2019. The Ministry of Finance is committed to ensuring that the USD 3.9 billion per year program achieves its goals [13]. A significant increase in funds will provide incentives for village community empowerment programs that allow villages to use village funds as capital to increase capacity and health promotion to handle stunting.

Most of the previous studies do not discuss village funds for capacity building and health promotions toward handling stunting as one of the priority programs for village funds. No literature empirically proves the capacity and capability of village governments to manage village funds. Accordingly, this study aims to identify how to influence village fund programs on the prevalence of stunting as a government intervention that is expected to provide solutions to various village problems, including health problems to handle stunting. This is undoubtedly inseparable from village government officials’ roles in maintaining the community’s commitment to development and community empowerment, mainly supported by authority and funding. It is an exciting phenomenon to research considering the village’s role as the smallest government administration unit and closest to the community, so it is expected that the existing problems are quickly resolved. This study is expected to contribute to existing knowledge of stunting by providing empirical evidence-based on data from all regions in Indonesia by looking at how village fund programs contribute to fighting against stunting.

This paper is organized as follows. The first section provides an overview of the importance of the research and its novelty. The second section presents a literature review, and the third section outlines the research methodology and data. The fourth section sets out our findings and discussion, followed by a conclusion and implications in the fifth section.

## Literature review

Numerous countries have implemented similar community-based development programs, such as village fund programs in Indonesia. For example, the KALAHI CIDSS program in the Philippines has increased consumption and employment [14]. Thailand, with the Thai Village Fund, showed the possibility of a small impact on reducing poverty or had no positive impact on the country’s poverty [15, 16]. Government budgets for the expenditure of goods and services, capital expenditures, conditional cash transfer (CCT) programs, and village fund programs (village funds) are various interventions that impact community welfare and economic growth.

Bokhari et al. [17] provide econometric evidence that the elasticity of infant and maternal mortality to government spending is negative, meaning that the higher government spending in the health sector, toddlers, and mothers’ death rate can be diminished. This implies that while economic growth is an essential contributor to health outcomes for developing countries, government spending in the health sector is equally crucial. Recognition of the synergistic impacts of spending on health and other sectors (e.g., water and sanitation, utilities, road networks, and education) is critical. Moreover, Berman and Ahuja [18] conducted a study specifically on government health spending in India by analyzing the latest health spending trends that had increased by 2–3 percent of GDP for over five years but were not yet able to meet the goals set. The study noted that the right fiscal targets for health spending should be based on plans to achieve the outcomes and resources needed to achieve them, most of which are still lacking. This shows that a large and sustained increase in government health spending requires more focused or priority programs and increases regional capacity to the smallest units in using existing resources for more effective health.

Furthermore, a study conducted by Leroy et al. [19] developed a model of the impact path of government spending through conditional cash transfer (CCT) programs on child nutrition. Through a review of evidence using the program theory framework, Leroy et al.’s [19] study suggested that the CCT program significantly improves a child’s anthropometrics but has minimal impact on micronutrients. These programs also have a positive effect on some of the results in the nutrition improvement pathway. They found a considerable gap in knowledge of CCT programs’ mechanisms in improving nutrition, indicating that further development of this matter is required. The government must have a more specific set of nutritional measures based on program theory.

According to the WHO framework released in 2013 for understanding the context and causes of stunting in children, many factors contribute specifically to stunted growth and development, including community and social factors, which are the only elements under the contextual determinant of child stunting within the framework [7, 20]. These community and social factors include political economy, health, health care; education; society and culture; agriculture and food systems; water, sanitation, and the environment. Given the diversity of geography and culture in Indonesia, the determinants of child stunting are most likely to differ geographically and politically [20].

This current study mainly focuses on analyzing the context of the political economy in influencing the prevalence of stunting. We develop a rationale based on tools and resources for nutrition capacity assessment as operational support in assessing the influence of village funds on the prevalence of stunting in the region. It was adapted from the United Nations (UN) Network for Scaling Up Nutrition (SUN UN Network) and Nutrition Capacity Assessment of Indonesia (NCAI) in 2018 [21] (see S1 Fig).

There are four fundamental concepts of NCAI. The first consists of policies, plans, and frameworks. This concept aligns with the program theory that village funds are sourced from the state budget and transferred directly to the village government in each district/city, requiring political commitment from the village head as a policy stakeholder. For example, a community empowerment program/activity plan adjusted to the priority program of using village funds set by the Ministry of Rural Affairs to cover sectors that are still lacking, including villages with high stunting cases. The second concept is that village fund programs can increase resources and infrastructures to be invested in health, including improving nutrition and nutrition for children and pregnant women to reduce the risk of stunting. This concept is inseparable from the role of human resources (village government apparatus) that must have adequate skills in carrying out action theories for large budget changes to be sourced from village funds.

The third concept is that evidence-based decision-making requires information, reporting, and dissemination systems related to village fund programs that focus on economic development and community empowerment activities. This can be illustrated from the village head/village’s preference in each district/city as a regional leader in the area, whether oriented to interests or needs, especially handling stunting. Empirical evidence provided by Martinez-Bravo (2014) shows that there is a difference in the tendency of elected officials to appoint officials. The tendency of the village head as a directly elected official by the community will pay more attention to the desires of his local constituents than the one appointed by the regent/mayor, who will tend their partiality to the ruler at the district/city level. Finally, the fourth concept is coordination and partnership, namely, coordination of nutrition action at all levels at the district level, including the involvement and collaboration of regional executives (village chief/*kadus*, RT chairmen, chairmen, and others). This also embraces the establishment of partnerships with relevant stakeholders, such as health centers, village midwives, and nutritionists (TPGs), to create integrated coordination and efficiency in the utilization of various resources, such as coordination between village governments and health centers in terms of more severe stunting mapping or a high incidence of chronic malnutrition. With continuous support and coordination that is well established among existing stakeholders, it is expected that the utilization of village funds can encourage the improvement of essential services and stunting prevention services, integration of nutrition counseling, clean water, and sanitation and social protection. This incorporates supporting the existence of village health posts/*posyandu*, the establishment of water and village sanitation organizations (BPPSPAM), and the establishment of playgroups, kindergartens, and childcare/PAUD (World Bank, 2020).

The priority use of village funds suggests that village fund programs vary based on the utilization categories. S2 Fig shows the scheme of priority utilization of village funds as stated in Regulation of the Minister of Villages No. 5/2015 that village funds prioritize two main objectives, village development and community empowerment. Capacity building and health promotion are parts of the goal of community empowerment. The village can carry out programs and activities to reduce malnutrition risk (stunting) and empower local communities for common welfare.

## Data and empirical methods

Data analysis in this study is based on the data collected from various sources. In the 2018 fiscal year, the Village Fund budget (DD) covers 434 districts/cities with 74,958 villages throughout Indonesia. The use of aggregate data is because stunting data at the *village* level are not yet available. This study utilizes cross-sectional data from a potential village survey (PODES) in 2014 and 2018 from the Central Statistics Agency. Meanwhile, stunting data in 2014 and 2018 were obtained from Basic Health Research (Riskesdas) of the Ministry of Health. The district/city population is then deployed to determine village funds per capita variables as the main data management in this study. The poverty rate and average length of women’s schooling are tracked from BPS. The use of two-year panel data in 2014 and 2018 conforms to Podes data published in that year. Other variable controls used in this study are the number of villages and the quality of health traced from BPS. Control of data is conducted using the information on village government apparatus and another financing source of data from the regional government budget (APBD) for health functions.

We estimated the model with a fixed effect (FE) to allow the presence of unobserved variables that are not all included in the model, that is, an interception that is not constant. Assuming that the α constant for each *i* and *t* is less realistic, these interceptions may change for each individual and time. In previous studies, fixed effect models have also been used by Cahyadi et al. [22] to examine the impact of CCT programs on stunting in children. Furthermore, the empirical models exploited to answer the questions in this study are as follows:

$$\begin{array}{c} {Stunt}_{it}=\alpha_{0}+\alpha_{1}{VFpc}_{it}+\alpha_{2}{educ\_kades}_{it}+\alpha_{3}{rapdes}_{it}+\alpha_{4}{VFxKades}_{it}+\\ \alpha_{5}{lnAPBDhealth}_{it}+\alpha_{6}{doktor}_{it}+\alpha_{7}{midwives}_{it}+\alpha_{8}{TPG}_{it}+{ALS}_{it}+\alpha_{9}{Povrate}_{it}+\\ \alpha_{10}\alpha_{11}{GDPpc}_{it}+\alpha_{12}{NV}_{it}+\gamma ddist+\varepsilon_{it} \end{array}$$ (1)

Notes:

*Stunt*_*it*_ = prevalence of stunting

*VFpc* = village fund budget per capita

*Educ_kades* = the average length of schooling of the village head is measured by transforming the ideal school time based on the highest education that is finished from elementary school to doctorate, then divided by the number of villages in one district/city

*Rapdes* = the average number of government apparatus other than the village head/measured by summing up all village apparatuses except the village head in one area and is divided by the number of villages in the same area

*VFxKades* = variable interaction of village fund per capita with the education of cadres

*lnAPBDhealth* = regional government budget (APBD) realization of district/city health function is included as a control variable because this variable is instrumental in separating the impact of village funds on health services

*Doctors Midwives*, *TPG* = health quality control in each region that is calculated based on the number of general practitioners, midwives, and nutrition support personnel’s vital roles and support in the village

*Povrate* = the poverty rate of a region

*ALS* = the average length of schooling that describes the level of education of women in the area

*GDPpc* = gross regional domestic product per capita

*NV* = number of villages to see the differences in the effect of village funds because the differences in the number of villages also affect the total village funds received in a district/city

*ddist* = dummy district/city

The *i* index refers to the districts/cities that are the research samples, the *t* index refers to the time period (years), *α*_0_ is the intercept, *α*_1,*dst*_ and γ are the estimated slope parameters, and *ε* is the *idiosyncratic error*.

The omitted variable may occur due to limited sources and data weaknesses aggregated from *village* to district/city level. Differences in economic and financial structure between districts/cities can lead to differences in the community’s socioeconomic conditions in all regions. Additionally, population density and variation between districts make it difficult to compare village funds’ average impact without creating equivalent units for the entire region.

## Results and discussion

Table 1 shows that the variations in village funds at the district level vary widely, depending on the number of villages and several other assessment indicators. The table shows a statistically considerable variation in the distribution of village funds among islands in Indonesia. Java Island receives a considerable amount of village funds, 33% of the total village funds, compared to other islands in Indonesia. Meanwhile, the regions outside Java acquire a relatively small amount of village funds, especially Bali-Nusa Tenggara, the islands of Maluku and Papua, and each receives 7%, 3%, and 9% of the total village funds disbursed nationally. In contrast, village funds per capita from regions on Java Island are much less than other regions outside Java; even Java only receives 12.5% of village funds per capita compared to Papua. This is due to the dense population on Java Island, leading to the smaller amounts received if calculated per capita.

**Table 1:** Share of number of villages and village funds in Indonesia for the year 2018.

Islands	Number of villages	Village funds (VF)*	*Share*	VF per capita (*IDR*)
Java	22,382	19,092,331,616	0.33	168,466
Non-Java	50,795	39,644,569,489	0.67	427,650
Sumatera	22,781	17,019,661,925	0.29	375,362
Bali-Nusa Tenggara	4,530	4,126,596,953	0.07	307,561
Kalimantan	6,566	5,230,981,024	0.09	430,655
Sulawesi	8,098	6,335,958,783	0.11	417,565
Maluku	2,190	1,695,202,008	0.03	621,910
Papua	6,630	5,236,168,796	0.09	1,343,379
**Total**	**73,177**	**58,736,901,105**	** **	** **

*VF in thousands of Rupiahs.

Source: Ministry of Finance 2017, *data processed*.

Table 2 presents descriptive statistics based on districts totaling 473 regencies/cities throughout Indonesia. The table shows that stunting in children occurs at an average of 34.39, with a standard deviation of 10.70, indicating that stunting is still very high in almost all observed areas. Village funds that become the primary variable in examining the effect on bound variables are average estimated value IDR 239,025 per capita in each district/city. Additionally, statistical figures show that an average of approximately 42 village heads in each district/city have completed their undergraduate studies from the bachelor’s to doctoral levels. In contrast, the average number of village apparatuses other than village heads statistically shows variations, with an average minimum of 4 people and a maximum of 127 people at Indonesia’s district/city level.

**Table 2:** Summary statistics.

Variables	Unit	Mean	Std.Dev.	Min	Max
*Dependent*:					
Stunting	*prevalence rate*	34.389	10.701	2.1	80.6
*Independent*:					
Village fund per capita	*million (IDR)*	.2390258	.5700222	0	11.36872
*Kades’ qualification* ‘ (average length of schooling)	*year*	12.269	2.218	.026	17.035
Average amount of village officials	*person*	24	21	4	127
APBD health function	*natural log*	18.811	.709	14.950	22.239
Doctors	*person*	97	143	2	1528
Midwives	*person*	334	246	1	1513
Nutritionists (TPG)	*person*	21	19	0	240
GDP per capita	*million (IDR)*	33.057	32.226	2.024	311.038
Number of villages	*area*	166	119	13	852
Law income societies	*person*	12.428	7.571	1.98	44.49
Average length of schooling (female)	*year*	7.473	1.767	.45	12.35
*N (Number of observations)*		946			

Source: Riskesdas, Podes, BPS and Ministry of Finance, *data processed*.

The estimation results in Table 3 show that village fund programs statistically significantly negatively influence stunting prevalence. The table indicates that the effect of increasing one million rupiahs per capita of village funds given to districts/cities tends to decrease the prevalence of stunting in the districts/cities by 1.96 points and is statistically significant at a 5% alpha. Previous studies [19, 22] have also focused on similar government programs, such as the family hope program (PKH), which aims to improve children’s nutrition and reach their full potential. The programs reported in these studies need to be evaluated to have more precise nutritional goals and require cumulative investment to show the decreasing impact on the prevalence of stunting [19, 22].

**Table 3:** The effect of village funds and the capacities of village apparatuses on stunting prevalence.

	(1)	(2)	(3)	(4)	(5)	(6)
	Stunt	Stunt	Stunt	Stunt	Stunt	Stunt
VFpc	-3.3172***	-2.6586***	-1.6451**	-1.6890**	-1.6475**	-1.9624**
	(0.7067)	(0.8867)	(0.7063)	(0.6980)	(0.6936)	(0.8180)
Educ_ village heads		-0.0490	0.1309	0.1095	0.1157	0.1007
		(0.4185)	(0.6138)	(0.6109)	(0.6123)	(0.6142)
Rapdes		-0.1199***	-0.0673**	-0.0722**	-0.0780***	-0.0765***
		(0.0198)	(0.0277)	(0.0281)	(0.0283)	(0.0286)
VFxVillage heads		0.0108	0.0308**	0.0286*	0.0262	0.0334*
		(0.0248)	(0.0156)	(0.0160)	(0.0166)	(0.0192)
lnAPBDhealth			-0.4675	-0.4065	-0.3596	-0.3138
			(1.5277)	(1.5366)	(1.4662)	(1.4763)
Doctors			-0.0157**	-0.0158**	-0.0175**	-0.0180**
			(0.0072)	(0.0072)	(0.0073)	(0.0074)
Midwives			0.0004	0.0006	0.0012	0.0006
			(0.0077)	(0.0078)	(0.0079)	(0.0080)
TPG			-0.0762***	-0.0765***	-0.0775***	-0.0772***
			(0.0283)	(0.0288)	(0.0290)	(0.0291)
Povrate				-0.4177	-0.4611	-0.4720
				(0.4394)	(0.4406)	(0.4420)
ALS					-4.0381*	-3.7984
					(2.3537)	(2.3995)
GDPpc						-0.0522
						(0.0595)
NV						0.0231
						(0.0314)
Constant	35.1817***	38.4427***	47.3653	51.8767*	80.7074**	76.4592**
	(0.3519)	(5.0737)	(30.1067)	(30.6605)	(36.3599)	(36.8122)
*Observations*	946	946	946	946	946	946
*R* ^*2*^	0.0446	0.1159	0.1472	0.1491	0.1547	0.1560
FE regencies/cities	Yes	Yes	Yes	Yes	Yes	Yes
Year FE	No	No	Yes	Yes	Yes	Yes

Standard errors in parentheses

* *p* < .1

** *p* < .05

*** *p* < .01.

The role of village officials in this study infers that village heads’ high academic qualification does not necessarily affect the decreasing prevalence of stunting. This might result from the village head’s preferences to allocate higher budgets for village funds to infrastructure development and physical expenditure as a benchmark for development. This indicates that health sector expenditure at the village level is very limited, which affects immediate actions to manage stunting. This may be due to the different preferences of the village head identified in the study as far as stunting is concerned, wherein the decision-making of local officials selected and appointed are influenced by interest factors [23]. On the other hand, Bokhari et al. [17] said that allocating more funds for health spending may not be effective if the government’s corruption level leads to small budgets on health care needs. Furthermore, Lewis [24, 25] states that unclear service assignments, fast-growing and relatively large budgets, inadequate public financial management procedures, and dubious control and accountability mechanisms raise particular concerns about potential corruptions at the local (village) level and a relatively fragile governance environment. The village head’s education estimates have not shown an impact on the decreasing number of stunting events in all districts/cities. This study denotes that the village head’s commitment to fighting against stunting in children is weak. In contrast to early findings Martinez-Bravo [26], we found that the increasing level of village heads’ education does not significantly improve community access to health services as one of the basic sectors in the community.

However, this study statistically proves a significant influence of other village apparatuses’ capacities, meaning that any increase in the average number of village apparatuses in one district/city will consistently reduce stunting prevalence. This implies that it is crucial to recruit a number of village apparatuses, such as village secretaries, technical implementers, regional executives, and other village employees, to support the organizational structure and work collaboratively in the village under the prevailing regulations in their specific areas. The fulfillment of the position is expected to support programs and activities that favor the health sector. This can be realized if there is good coordination and cooperation between existing elements in supporting health programs or handling stunting, including coordination with relevant stakeholders such as health centers in their respective regions. The lessons from Peru to successfully reduce stunting for children are as follows: first, establish political commitment, cooperation, and coordination; second, smarter policies from policymakers focusing on evidence, incentives, and outcomes; and third, change goal-oriented behavior, especially stunting handling [8].

The quality of health services in each region entails equal attention. In addition, it is vital to gain health workers’ assistance with educating mothers about home and environmental cleaning and to enhance community awareness to ensure children’s health [27]. The incidence of stunting is associated with determinants of people’s socioeconomic status in Indonesia [20]. The role of women’s education levels in this study differs from previous studies that showed that maternal education exerts a considerable influence on stunting prevalence [7]. This is due to the limited data used in regression, namely, the average data of women’s education in one district/city. In contrast, previous studies [5, 7, 20] employed maternal education level data because not all women have become mothers. Simultaneously, the poverty level of a district/city does not affect the prevalence of stunting. This may be because the change in income alone will not automatically solve the problem of nutrition in children, and the incidence of stunting is surprisingly also very high even in the quartile of the richest people [5].

The regional government budget (APBD) on health functions has not significantly influenced the prevention of stunting prevalence. This is also in line with a previous study in India, which found that spending on health functions has not been entirely based on handling and preventing stunting and the capacity of health workers resources that need to be improved [18]. Additionally, Gupta et al. [28] maintain that government spending in the health sector affecting health services and death rates remains relatively weak. The possibility of more allocation of health funds is directed to alleviate diseases and still very little to fulfill children’s nutrition. Then, GDP per capita shows the results of estimates that have not affected the betterment of children’s chronic malnutrition, and this confirms previous studies that found that increased public spending aimed at combating malnutrition is not predominantly targeted to meet the needs of the inferior [9]. The estimated GDP per capita of districts/cities in this study shows that regardless of the areas with low and high GDP, the prevalence of stunting remains. This means that although districts or cities on the island of Java have a large GDP per capita, some children are still identified as having growth issues.

Furthermore, the differences in village fund relations occurring in Java and non-Java show that village funds influence stunting prevalence in districts/cities on the non-Java islands (see Table 4). However, there is a nonsignificant effect in districts/cities on Java Island. This is alleged because the low receipt of village funds per capita in Java is the leading cause; in addition, the sample on the island of Java is much less than the sample outside Java Island, approximately one-third of the non-Java sample. The result of regression on the island of Java looks insignificant because village funds’ needs are higher in Java. If calculated per capita, the regions on the island of Java receive fewer village funds per population than areas outside Java.

**Table 4:** Village funds, village apparatus capacities and stunting in Java and outside Java.

	(1)	(2)
	Stunting	Stunting
Village fund per capita	0.7897	-1.8443**
	(7.9108)	(0.8776)
Education of head of	-0.0866	0.1313
*village*	(0.5864)	(0.7298)
Average village apparatuses	-0.0655***	-0.1964***
(other than the head of *village*)	(0.0231)	(0.0536)
Constant	-82.9148	64.7697*
	(59.4419)	(34.7574)
*Observations*	222	724

Standard errors in parentheses

* *p* < .1

** *p* < .05

*** *p* < .01.

The results of the estimates show that any increase in the average number of village apparatuses other than the village head/in a district/city will be able to affect the reduction of the prevalence of stunting compared to the level of education of village heads, both in Java and outside Java. This implies that the existence of apparatuses other than the head of village, such as the head of hamlet, head of RT, head of RW, Linmas, and other apparatuses, becomes very important, both in Java and outside Java. Other village apparatuses are closely attached to the community because of their quick access to information related to various environmental problems, including information on communities associated with stunting.

The majority of the village’s work is in agriculture. Thus, it is also necessary for the village head to use village funds to build facilities and agricultural development infrastructure. It will rely upon on-farm technology and environmental efficiency to increase farmer productivity, community income, and welfare. Previous studies have suggested the need to assess ecological efficiency in creating a sustainable agricultural system to avoid potential negative factors from technological advances, as in the case of Latin America and the Caribbean [29]. Furthermore, by testing agricultural productivity growth in 14 countries, the results of those studies showed an increase of total factor productivity annual growth of 1.5%, with efficiency changes (or catch-ups) contributing 0.1% per year [30]. If the village fund can accomplish the community’s welfare, it may also decrease the stunting rate in that village.

## Conclusion and policy implications

The overarching objectives of village fund programs include developing and empowering village communities. Stunting risk control is part of community empowerment because it aims to increase capacity and health promotions to the local community. The study aims to examine the impact of village funds on the prevalence of stunting in Indonesia. The study’s findings indicated that village fund programs could significantly reduce the prevalence of stunting throughout Indonesia, except for areas on the island of Java, due to variations in village funds and populations. This is in line with the initial allegations of this study, which estimated that village funds affect the prevalence of stunting, especially in areas that secure greater village funds per capita and have a better village apparatus capacity. Village funds per capita on Java Island are much smaller than per capita village funds outside Java because Java is Indonesia’s most populous island. Hence, the estimated impact of funding (village funds) on the prevention of stunting is lower and may have no impact.

Nonetheless, there is no evidence that local leaders’ role affects the incidence of stunting in their areas. However, we found evidence that village apparatuses other than the village head are statistically significant in reducing the prevalence of stunting throughout Indonesia. Additionally, there are differences in local officials’ preferences; for instance, the village heads elected by the community will usually tend to accomplish the community’s will as part of their accountability to voters. In contrast, the *village heads* appointed by the regional head will tend to be more influenced by the interests of the officials who appoint them.

The implication of this study is the size of the village budget rolled out by the government in encouraging development and community empowerment in the region (village) in addressing the challenges of tackling the prevalence of stunting to support national priorities in the health sector. There needs to be a sound policy from the village head in each district/city to design and budget the village fund to contribute to the needy individuals, especially those associated with stunting. Furthermore, this study shows the importance of actively supporting other government apparatuses at the RT/RW (smallest unit in the village) level to become resilient assets to handle stunting. Furthermore, it is important to build and increase awareness of village heads on health activities, especially early prevention of stunting.

It is plausible that several limitations may have influenced the results obtained. First, we have difficulties accessing data of stunting prevalency and village funds at the village level that are publicly available; as a result, we have to use the aggregated data at the district/city level. Second, little is known about the research on village-level funds and the quality of local officials, especially at the village level; therefore, it is possible to ignore the variable deviations that lead to the prevalence of stunting. There is also a possibility of endogeneity related to other factors affecting village funds and differences in the preferences of village heads based on their genders and preferences on the issues of stunting because of the difficulties in detecting them through sufficient data

## Supporting information

S1 Fig The mechanism for using village funds to prevent stunting. (DOCX)

S2 Fig The priority of village funds utilization. (DOCX)
