# Supplementary figures and images for: Understanding the role of village fund and administrative capacity in stunting reduction: Empirical evidence from Indonesia
Source: PLoS One. 2022 Jan 28;17(1):e0262743. doi: 10.1371/journal.pone.0262743 (PMC8797224; doi:10.1371/journal.pone.0262743)

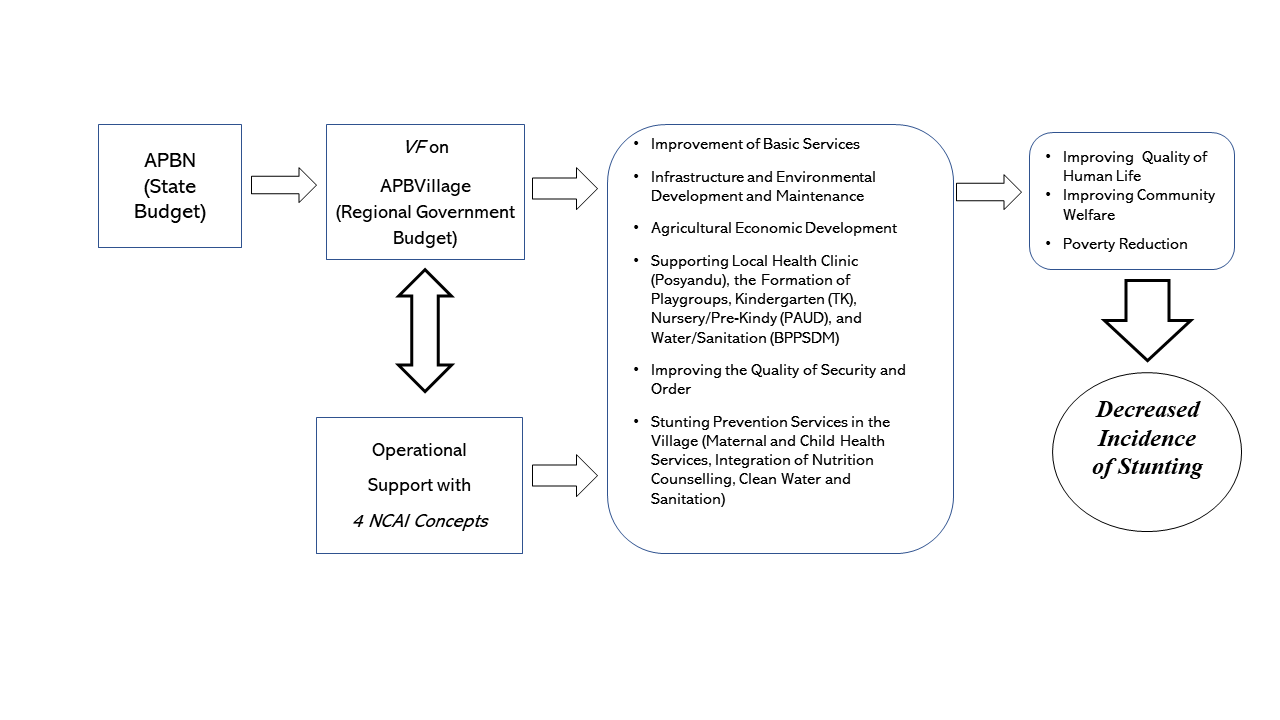


**S1 Fig. The Mechanism for Using Village Funds to Prevent Stunting**

Supplement: S1 Fig — (DOCX) [file pone.0262743.s001.docx]
