# Supplementary material for: Understanding the role of village fund and administrative capacity in stunting reduction: Empirical evidence from Indonesia
Source: PLoS One. 2022 Jan 28;17(1):e0262743. doi: 10.1371/journal.pone.0262743 (PMC8797224; doi:10.1371/journal.pone.0262743)
Supplement: S2 Fig — (DOCX) [file pone.0262743.s002.docx]

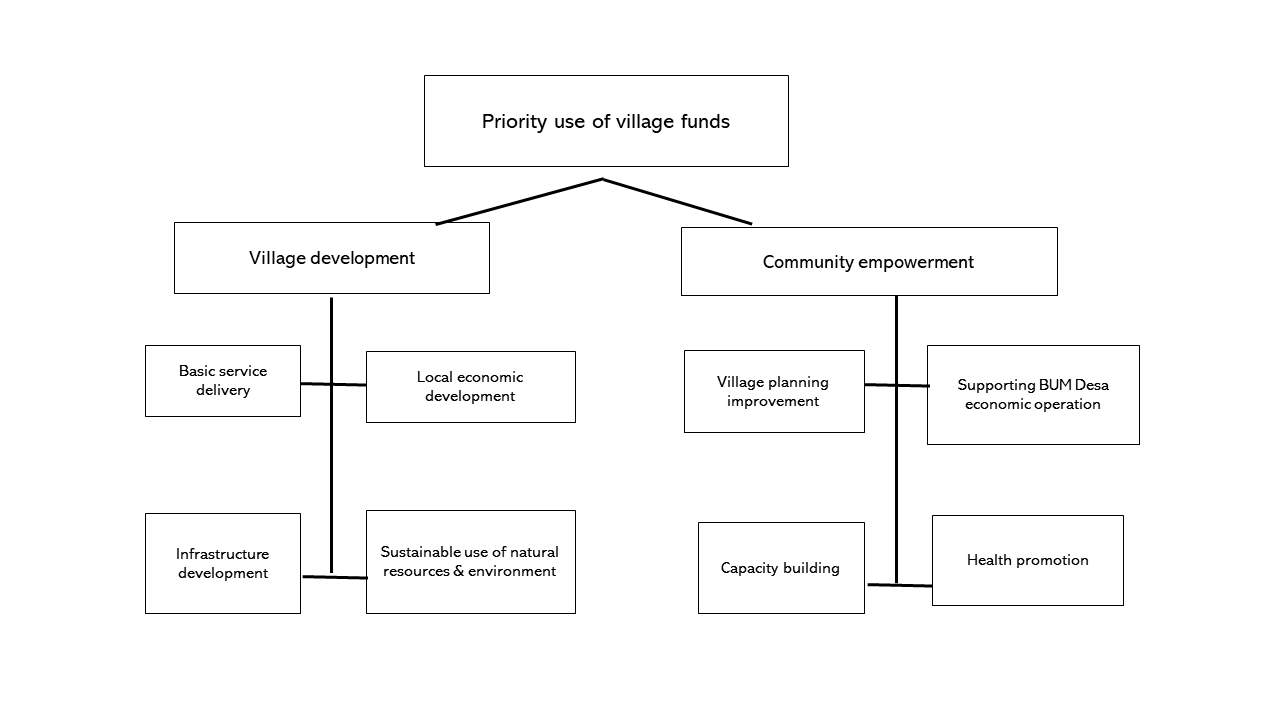


**S2 Fig. The Priority of Village Funds Utilization**

Source: Regulation of the Minister of Villages No. 5/ 2015
